# A proof-of-concept minimum data set for older care home residents: what value does this add?

**DOI:** 10.23889/ijpds.v9i5.2850

**Published:** 2024-09-18

**Authors:** Freya Tracey, Elizabeth Crellin, Kaat De Corte, Therese Lloyd, Richard Brine

**Affiliations:** 1 The Health Foundation

## Objective

Developing resources And minimum data set for Care Homes’ Adoption (DACHA) is a four-year NIHR-funded project to establish what data need to be in place to support research, service development and innovation uptake in care homes for older people. An aspirational minimum data set (MDS) specification was identified, which included data from several sources and pilot variables.

## Approach

767 care home residents (with a valid NHS number recorded) across three integrated care systems consented to linkage of their care home record with administrative health and social care records from NHS England. Data were linked using pseudonymised NHS number. Variables were derived to summarise healthcare utilisation.

Where variables are sourced from more than one data set, expert opinion on quality across these data sets informed hierarchical selection. We reported descriptive statistics including missingness across each of the >300 variables in the MDS, with information on how these were accessed and derived, where appropriate. Descriptive summaries were informed by stakeholder engagement and cover ambulance and emergency department utilisation.

An ongoing DACHA PPIE panel contributed to MDS development through sharing opinions on data access and linkage, and best sources of information across different domains.

## Results

Analysis is in progress. All results will be in pre-print publication by 31 April 2024.

## Conclusions

The MDS constructed gives rich insights on healthcare utilisation and resident needs, with opportunity to understand how these interrelate.

## Implications

As a proof-of-concept study, the findings of this research will inform national decision making on future linked care home data sets.

